# A Feasibility Study of Floatation-REST for Fatigue: An Idea That Was Worth Floating

**DOI:** 10.1192/bjo.2024.261

**Published:** 2024-08-01

**Authors:** James Todd, Hugo Critchley, Michael Cordova, Jessica Eccles, Alessandro Colasanti

**Affiliations:** 1 Sussex Partnership Foundation Trust, Brighton, United Kingdom; 2 Brighton and Sussex Medical School, Brighton, United Kingdom; 3 Floatation Tank Association, Bedford, United Kingdom

## Abstract

**Aims:**

Floatation-REST (restricted environmental stimulation therapy) has shown promising potential as a therapeutic intervention in psychiatric conditions such as anxiety and anorexia nervosa. We speculate that the sensory deprivation might act as a kind of interoceptive training. Within our lab, interoceptive trait prediction error has been used to predict states of anxiety in autistic adults. There is also emerging research conceptualising interoceptive mismatches potentially playing a role in fatigue. Our aim was to run a feasibility study assessing the tolerability of Floatation-REST for participants with disabling fatigue. We also aimed to establish the feasibility of gathering data on mechanistic measures, such as heart rate variability (HRV) and interoception, during floatation.

**Methods:**

Participants were recruited via online advertisements and were screened to check they scored at least 36 on the Fatigue Severity Scale (FSS). Pertinent medication changes and previous float experience within the last 6 weeks were amongst the exclusion criteria. Baseline measures included: Modified Fatigue Impact Scale (MFIS); Body Perception Questionaire; hypermobility questionnaire and Tellegen Absorption Scale. Participants completed four 90 minute sessions of floatation-REST across a 2–6 week period with 1 week of ecological momentary sampling (EMS) before and after. Immediate pre and post float measures included testing interoceptive sensibility, accuracy and awareness. HRV was measured during floatation. Change in energy was measured by retrospective subjective assessment, changes in validated fatigue scales and EMS.

**Results:**

Baseline MFIS scores (median = 67.5; range = 55–77) indicated a high degree of severity of participant fatigue. 15 participants were recruited to the study. 13 participants started the float intervention and 11 completed all four sessions. No drop out was due to poor tolerability. Most adverse events were mild, expected and related to the pre/post float testing. HRV data was successfully captured throughout all sessions. Participant surveys described improvements in energy levels, sleep and relaxation and 73% “strongly agreed” to an overall positive effect. Furthermore, both statistically and clinically significant reductions were noted in the mean FSS scores (56.9 to 52.6; p = 0.044) and the MFIS scores (67.0 to 56.4; p = 0.003). Detailed energy assessment was obtained by EMS with 37 to 86 data points per participant.

**Conclusion:**

Floatation-REST appears to be a feasible intervention for people with severe fatigue. EMS, HRV data, interoceptive data and other measures were reliably recorded. Reported subjective benefits were supported by an improvement in objective fatigue scores, though the lack of a control group makes these improvements speculative at present.

